# Population structure of *Desmophyllum pertusum* found along the United States eastern continental margin

**DOI:** 10.1186/s13104-024-06977-4

**Published:** 2024-10-29

**Authors:** Alexis M. Weinnig, Aaron W. Aunins, Veronica Salamone, Andrea M. Quattrini, Martha S. Nizinski, Cheryl L. Morrison

**Affiliations:** 1 US Geological Survey, Eastern Ecological Science Center, Leetown Research Laboratory, Kearneysville, WV USA; 2 Department of Invertebrate Zoology, National Museum of Natural History, Smithsonian Institution, Washington, DC USA; 3 National Systematics Laboratory, Office of Science and Technology, NOAA Fisheries, Washington, DC USA

**Keywords:** Population connectivity, RADSeq, Deep-sea, Coral, Western north Atlantic Ocean

## Abstract

**Objective:**

The connectivity and genetic structuring of populations throughout a region influence a species’ resilience and probability of recovery from anthropogenic impacts. By gaining a comprehensive understanding of population connectivity, more effective management can be prioritized. To assess the connectivity and population genetic structure of a common cold-water coral species, *Desmophyllum pertusum* (*Lophelia pertusa*), we performed Restriction-site Associated DNA Sequencing (RADseq) on individuals from nine sites ranging from submarine canyons off New England to the southeastern coast of the United States (SEUS) and the Gulf of Mexico (GOM). Fifty-seven individuals and 3,180 single-nucleotide polymorphisms (SNPs) were used to assess genetic differentiation.

**Results:**

High connectivity exists among populations along the SEUS, yet these populations were differentiated from those to the north off New England and in Norfolk Canyon along the North Atlantic coast of the United States, as well as those in the GOM. Interestingly, Norfolk Canyon, located just north of North Carolina, and GOM populations exhibited low levels of genetic differentiation, corroborating previous microsatellite analyses and signifying gene flow between these populations. Increasing sample sizes from existing populations and including additional sampling sites over a larger geographic range would help define potential source populations and reveal fine-scale connectivity patterns among *D. pertusum* populations.

**Supplementary Information:**

The online version contains supplementary material available at 10.1186/s13104-024-06977-4.

## Introduction

Understanding population connectivity is vital to the development and implementation of effective management and conservation efforts, especially in the wake of ongoing environmental change. Assessing population connectivity in deep-sea environments is challenging, given the inherent difficulty of collecting samples in these locations. Contemporary genomic analyses can reveal patterns of population structure across depth and geographic distance at finer scales than other genetic approaches such as microsatellites [1]. Additionally, genomic-level analyses can support taxonomy and potentially reveal cryptic species, even within well-studied groups [2].

Marine resource managers require baseline information about an ecosystem before implementing regional conservation measures or utilization of natural resources. Baseline information at an ecosystem scale in the deep sea can consist of species identifications, biodiversity assessments, food-web dynamics, and evaluation of genetic connectivity between surrounding areas. Historically, ecosystem-level assessments have been difficult to conduct in the deep sea (> 200 m) due to technical constraints. With ongoing advances in both ship- and shore-based technologies, scientists now have more access to deep-sea ecosystems to collect, analyze, and synthesize baseline information, advancing our understanding of these habitats. These benthic ecosystems include cold-water coral (CWC) habitats, which are distributed throughout the world’s ocean, and frequently found on continental slopes, submarine canyon walls, and seamounts [3, 4]. Although the collection of CWC population data continues to increase, large knowledge gaps still exist, even for the well-known CWC, *Desmophyllum pertusum* (formerly *Lophelia pertusa*).

Scleractinian CWCs like *D. pertusum* form complex reef structures that increase habitat heterogeneity and thus contribute to an overall increase in regional biodiversity. These structures can be thousands to millions of years old, underscoring the long-term contributions of CWCs to deep-sea ecosystem health [4–10]. CWCs also have reproductive methods and larval phases that are conducive to long-range dispersal, resulting in complex genetic connectivity patterns. For instance, *D. pertusum* are gonochoric (separate sexes), periodic broadcast spawners with larvae that migrate upwards for the first five weeks, and then shift downward with a settlement period that can last over 50 days [11, 12]. Thus, understanding the connectivity patterns among existing *D. pertusum* populations is critical to effectively managing these CWC habitats and the ecosystem they support.

Past studies in *D. pertusum* have revealed discrepancies in long and short-range connectivity patterns. Based on 400 samples genotyped at eight microsatellite DNA markers, genetic discontinuity among *D. pertusum* populations was observed on a regional scale with four distinct genetic groupings Gulf of Mexico (GOM), southeastern coast of the United States (SEUS), New England Seamounts, and eastern North Atlantic Ocean, corresponding to defined ocean regions [13]. However, when samples from the mid-Atlantic canyons (including Norfolk Canyon) were analyzed at fourteen microsatellite loci, these populations were more closely related to the GOM than the neighboring SEUS [14, 15]. To investigate these findings further, we sampled individuals (68% newly sampled for this study, 32% from previous studies) from populations along the SEUS, GOM, New England canyons, and Norfolk Canyon; generated a set of genome-wide informative markers through restriction-site associated DNA sequencing (RADSeq); and used single nucleotide polymorphisms (SNPs) to provide finer-scale resolution of the levels of genetic diversity and gene flow among these regions.

## Methods

### DNA extractions and sequencing

DNA was extracted from 157 *D. pertusum* individuals collected over a 10-yr period during seven research cruises. Age and quality of tissue varied; therefore, several DNA extraction methods were used to obtain the quality and quantity of samples required for RADSeq. Methods included multiple extraction kits (Qiagen PureGene, the Qiagen DNeasy Blood & Tissue Kit (Hilden, Germany), and a modified CTAB method) [16] (Additional file 2). DNA was normalized to 20 ng/µl in 50 µl and sent to for library prep and RADSeq (Floragenex; Beaverton, OR). DNA libraries were constructed using the 6-cutter PstI and Mse1 enzymes, followed by sequencing 100 bp SE reads on an (Illumina HiSeq4000; University of Oregon’s Genomics and Cell Characterization Core Facility lab).

### Sequence processing and bioinformatics

Sequencing data from 157 *D. pertusum* samples were demultiplexed and assembled using ipyrad v.0.9.81 in *reference* mode; default parameters were used in the assembly [17]. A recently assembled and annotated *D. pertusum* (*Lophelia pertusa*) genome was used as reference [18]. Following the assembly, loci and SNP information were recovered in a variant call format (vcf) file and then filtered using vcftools v.0.1.16 [19]. After removal of samples with > 90% missing data, the remaining 57 samples were processed for further analysis (Additional file 1, filtering details described in Additional file 2).

Genetic diversity statistics were calculated in R (v.4.1.1) using the packages *adegenet* and *hierfstat* [20, 21]. Individuals were assigned to populations based on the collection sites. Mean observed heterozygosity (H_O_), mean expected heterozygosity (H_E_), overall gene diversity (H_t_), and the inbreeding coefficient (F_is_), were calculated for the entire dataset as well as for each population. Pairwise F_ST_ were calculated between the assigned populations according to Weir and Cockerham (1984) and plotted using the *ggplot2* package [22, 23]. The API: ipyrad-analysis toolkit was used to generate a principal components analysis (PCA) and to run STRUCTURE v.2.3.4 [17, 24] (Additional file 2).

## Results

From the 157 *D. pertusum* samples processed, 144,682 loci (57% of prefiltered loci) were assembled. After subsequent filtering with vcftools, 57 samples and 3,180 unlinked SNPs were included in further analyses.

Genetic differentiation among *D. pertusum* samples was evaluated for all sample sites except for Pea Island Seep (off North Carolina), since a single individual was sampled from this site. The diversity statistics suggest low heterozygosity among the sampled individuals (Supplementary Tables 1 & 2). Overall, inbreeding was relatively high (F_IS_ = 0.726), however, inbreeding coefficients were generally lower at each site (F_IS_ = 0.383–0.596) (Supplementary Table 3). A Wahlund effect could be contributing to the apparent heterozygote deficiency and elevating the F_IS_ values [25].

**Fig. 1:**
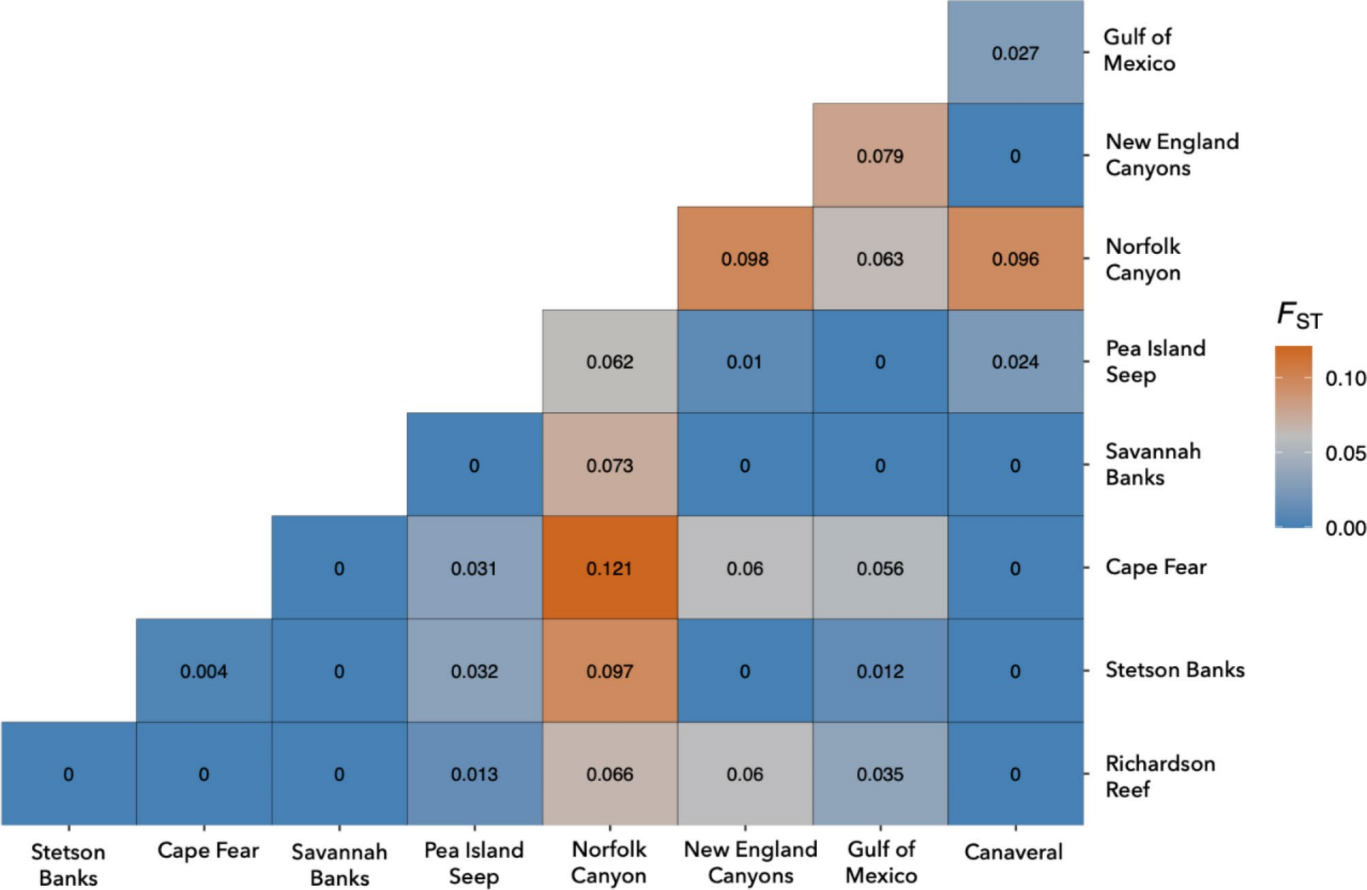
Pairwise F_ST_ values for *Desmophyllum pertusum* samples (*n* = 57) collected from nine sites. For values < 0, a 0 was applied. Analysis is based on 3,180 unlinked SNPs

Differentiation between sample sites was further analyzed using a Pairwise F_ST_ analysis. Pairwise F_ST_ values between the eight sample sites ranged from 0 to 0.121 (Fig. 1). Genetic differentiation was detected between many of the sites, with the highest overall F_ST_ values involving comparisons with Norfolk Canyon, ranging from 0.062 (Pea Island) to 0.121 (Cape Fear). Pairwise F_ST_ values were also high for comparisons involving the New England canyons (e.g., Norfolk Canyon = 0.098, GOM = 0.079). However, F_ST_ values were surprisingly low for comparisons between the New England canyons and several sites along the SEUS (Cape Fear, Stetson Bank, Savannah Banks, Richardson Reef, and Canaveral). Minimal genetic differentiation was detected between the SEUS sites (F_ST_ range 0–0.004), suggesting high connectivity within the region. Additionally, isolation by distance and isolation by depth analyses were conducted but there was no evidence of isolation by either parameter (distance: R^2^ = 0.255, *p*-value = 0.217; depth: R^2^=-0.009, *p*-value = 0.457) (Supplementary Fig. 1).

**Fig. 2:**
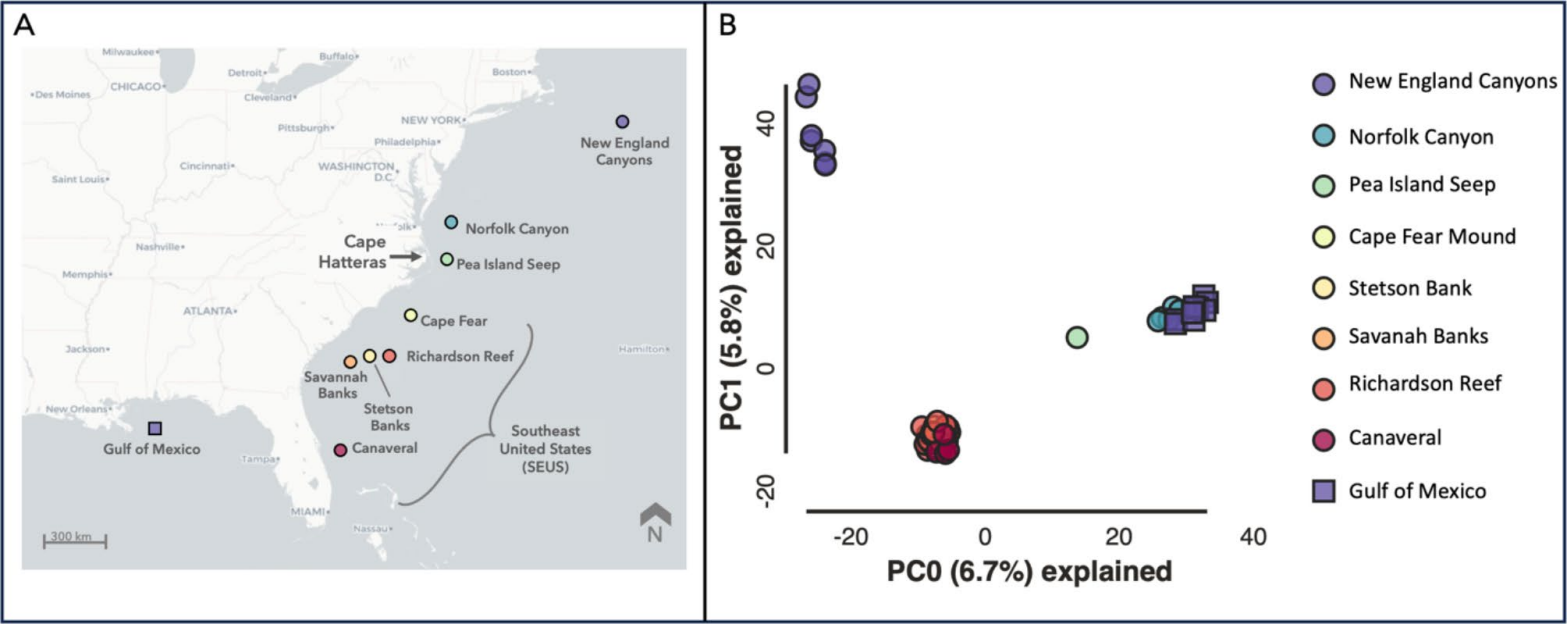
(**A**) Map of sampling sites. (**B**) Principal Coordinate Analysis (PCA) plot of genetic differentiation among *Desmophyllum pertusum* samples (*n* = 57) collected across nine sites. Analysis based on 3,180 unlinked SNPs

**Fig. 3:**
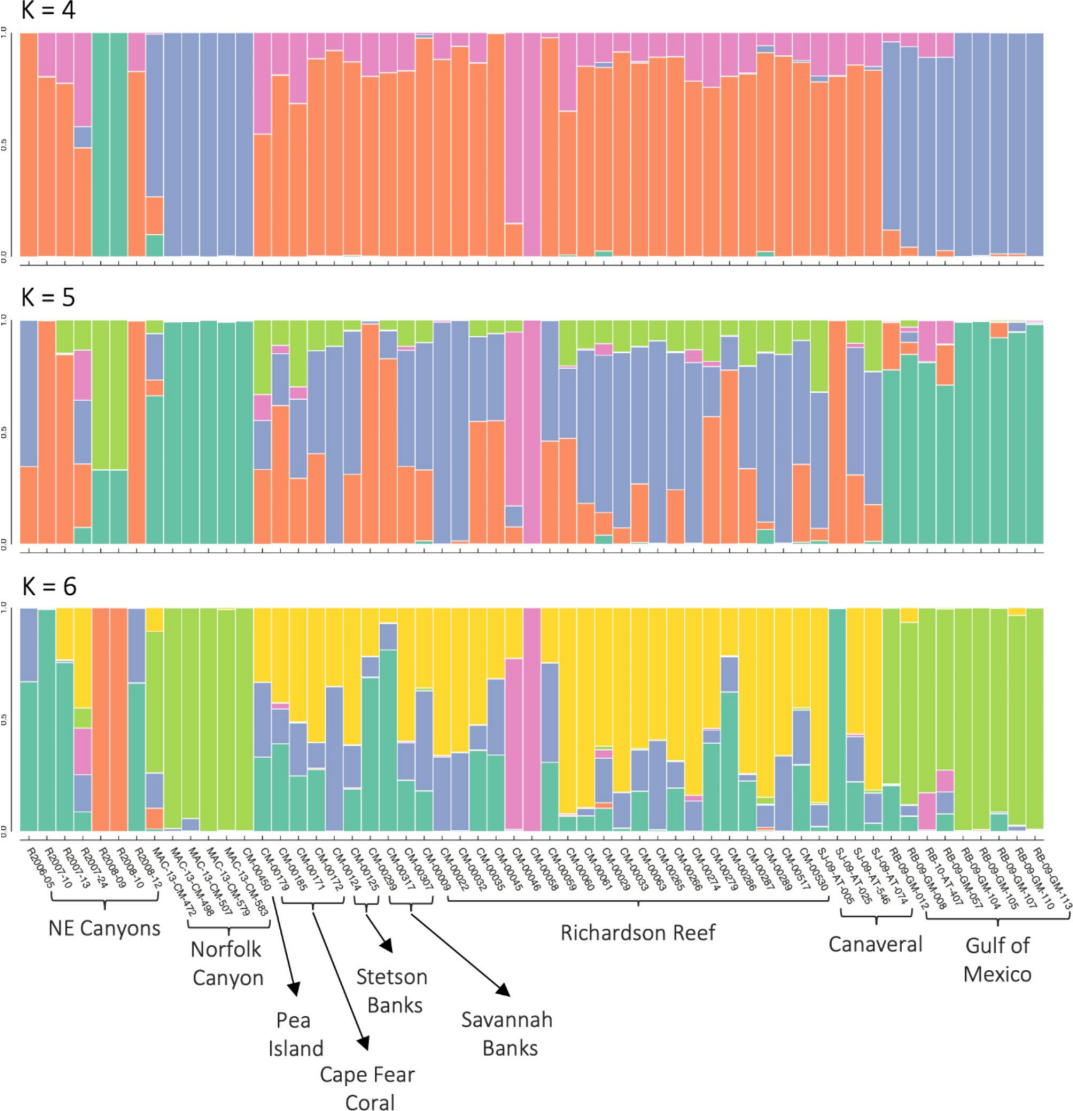
STRUCTURE results (K = 4, 5, 6) for *Desmophyllum pertusum* samples (*n* = 57) from nine sites. Each bar represents one sample and analysis is based on 3,180 unlinked SNPs. The Southeast United States (SEUS) populations are Cape Fear, Stetson Banks, Savannah Banks, Richardson Reef, and Canaveral

A Principal Component Analysis (PCA) based on genetic similarity identified a population structure of four genetic clusters (Fig. 2). These included: (1) the SEUS sites, (2) Norfolk Canyon and the Gulf of Mexico, (3) the New England canyons, and (4) the Pea Island sample. The first two PCA axes explained 6.7% and 5.8% of the variation. The STRUCTURE analysis, however, indicated that six genetic clusters were most likely (*K* = 6, Fig. 3, Additional file 2). In addition to the four populations demarked by PCA, STRUCTURE also indicated that two individuals from the New England canyons (R2008-09 and R2008-10) and two individuals from Richardson Reef (CM-00046 and CM-00058) formed separate clusters and appear to be derived from independent unsampled populations (orange and pink bars, respectively). At K = 6, individuals from Norfolk Canyon, Pea Island, and the GOM shared common genetic ancestry (lime green bars, Fig. 3), while sites along the SEUS exhibited admixture of several unsampled populations (turquoise, purple, yellow bars, Fig. 3.). While both the PCA and the STRUCTURE analyses grouped Norfolk Canyon and GOM samples and the SEUS samples as clusters, the STRUCTURE analysis indicates that most of the New England canyons samples appear to be a different proportion of the same core ancestral populations as the SEUS samples.

## Discussion

The ocean circulation of the western North Atlantic Ocean impacts larval dispersal, settlement, and overall connectivity of benthic populations [26, 27]. Utilization of thousands of genomic SNP markers for *D. pertusum* suggest regional genetic structuring concordant with pervious microsatellite analysis, yet SNP results provide finer detail about the likelihood that several unsampled populations are influencing the genetic structure of these regions, especially in the SEUS [13, 14, 16, 28]. Regional genetic structuring in *D. pertusum* is in agreement with previously documented coral-defined biogeographic provinces [14].

Our analyses suggest high connectivity amoung the SEUS sites (Cape Fear, Stetson Bank, Savannah Banks, Richardson Reef, and Canaveral). The PCA, pairwise F_ST_ values, and STRUCTURE analysis indicated that the SEUS sites were genetically similar to each other and constitute one population along the Blake Plateau. The STRUCTURE analysis indicated that at higher levels of *K* (e.g., *K* = 5–6), populations from the SEUS appeared more genetically admixed than was shown in previous analyses based on microsatellites. The SEUS populations, however, were differentiated from populations to the north, (Norfolk Canyon, Pea Island, the New England canyons) and from the GOM.

The New England canyons and Norfolk Canyon are north of Cape Hatteras, North Carolina, a known north-to-south biogeographic break in many shallow water marine taxa. Additionally, Cape Hatteras was recently proposed as a biogeographic break for a deep-sea glass sponge, *Vazella pourtalessi*, indicating that this feature may act as a dispersal barrier for other deep-sea taxa as well [26, 27]. The STRUCTURE analysis indicates that the SEUS populations are more genetically similar to the New England canyons than Norfolk Canyon. This genetic similarity could be a result of the turbulent hydrography caused by the meeting of several major currents in the area (Gulf Stream, the Labrador Current, or the Deep Western Boundary Current (DWBC) [29, 30]. The DWBC flows southward along the western boundary of the Gulf Stream from the Labrador Sea to the equator. A portion of the DWBC flows westward, inshore of the Gulf Stream before encountering the deep Gulf Stream near Cape Hatteras. Portions of the deeper DWBC waters flow underneath the Gulf Stream and continue in a south-westward orientation and could be contributing to the genetic diversity in populations of *D. pertusum* found along the SEUS and the relative genetic similarity to the New England canyons.

Interestingly, *D. pertusum* in Norfolk Canyon was genetically more similar to those from the GOM than populations in closer proximity, such as those off New England or the SEUS; a pattern also detected with microsatellite data [15]. While hydrodynamic connections with the GOM are not obvious, intrusions of Gulf Stream waters occasionally move onshore and may facilitate the migration of marine species to the Mid-Atlantic Bight (MAB) [27, 31]. Such onshore intrusions likely influence long-distance dispersal of several fishes, such as bluefish (*Pomatomus saltatrix)* [32] and the American eel (*Anguilla rostrata*) from the Sargasso Sea [33]. Similarly, GOM and MAB cold-seep mussels (*Gigantidas childressi*) are not genetically differentiated [34], suggesting on-going connectivity between these regions. Alternatively, additional populations of *D. pertusum* may exist in the western Atlantic in closer proximity to the MAB (e.g., Bahama Banks or the Caribbean) and may act as steppingstones for dispersal between the GOM and MAB. Notably, roughly half of the water transported through the Florida Current (southern portion of the Gulf Stream) is south Atlantic water that has been advected through the Gulf of Mexico and the Caribbean from the North Brazil Current [29]. The integration of fine-scale genomic population data with high-resolution oceanographic circulation models could yield valuable information about the distribution of source and sink populations of *D. pertusum* in the western North Atlantic, providing essential information for management and conservation of these ecosystems.

The relatively high inbreeding coefficient (F_IS_) values could be caused by a Wahlund effect, which typically occurs when the true scale of an organism’s population structure is unknown, resulting in unequal sampling across all populations [25]. However, significant inbreeding has been detected in many populations of this species previously [13, 14, 35]. Prevalence of asexual reproduction (fragmentation) during reef development and the longevity of individual clones are suggested causes for high inbreeding coefficients in this species and may influence patterns of population structuring [35, 36].

### Limitations

While these are encouraging results, a note of caution involving small sample sizes for several *D. pertusum* populations is warranted. Accuracy in defining population differentiation requires larger sample sizes (> 20 individuals per site) [37]. Similarly, the high quality and quantity of DNA required for RADseq can reduce the number of useable samples, particularly if sample collection occurs over decades, given the logistical difficulties of collecting deep-sea taxa. Obtaining high quality DNA from older samples was problematic, resulting in fewer samples included in our analyses.

Inclusion of samples from other regional populations of *D. pertusum* would also help to clarify the sources and sinks of larvae within and between regions. Notably, the Caribbean, a historically under-sampled region for *D. pertusum*, could be a source of larvae for populations along the SEUS. Similarly, more northerly populations (i.e., Greenland or the Laurentian Channel) could contribute larvae as they are carried south by the Labrador Current or DWBC. Nevertheless, our study provides insight into the complex population structure of an ecologically significant coral species and suggests that hydrography around Cape Hatteras has the potential to impact population structure in deep-sea organisms, as well as shallow-water taxa.

## Electronic supplementary material

Below is the link to the electronic supplementary material.

Supplementary Material 1

Supplementary Material 2

Supplementary Material 3

Supplementary Material 4

Supplementary Material 5

Supplementary Material 6

## Data Availability

Raw data can be found at NCBI GenBank BioProject number PRJNA1027916 and analysis steps can be found at https://gitlab.com/aweinnig/lophelia_radseq. By agency policy, the raw data have also been made available at the USGS’s ScienceBase portal at 10.5066/P145JOIO.
