# Supplementary material for: Population structure of Desmophyllum pertusum found along the United States eastern continental margin
Source: BMC Res Notes. 2024 Oct 29;17:326. doi: 10.1186/s13104-024-06977-4 (PMC11520793; doi:10.1186/s13104-024-06977-4)

Additional File 2

Title: Population structure of *Desmophyllum pertusum* found along the United States eastern continental margin

Alexis M. Weinnig^1^, Aaron Aunins^1^, Veronica Salamone^1^, Andrea M. Quattrini^2^, Martha S. Nizinski^3,2^, and Cheryl L. Morrison^1^

^1^US Geological Survey, Eastern Ecological Science Center, Leetown Research Laboratory, Kearnesville, WV USA

^2^ Department of Invertebrate Zoology, National Museum of Natural History, Smithsonian Institution, Washington, DC USA

^3^ National Systematics Laboratory, Office of Science and Technology, NOAA Fisheries, Washington, DC USA

**Any use of trade, product, or firm names is for descriptive purposes only and does not imply endorsement by the U.S. Government.**

*DNA extractions and sequencing*

The DNA from each sample was quantified using the Qubit dsDNA BR Assay Kit (ThermoFisher) and assessed using a 2.0% agarose gel electrophoresis with GelRed® DNA stain (Biotium, Fremont, CA). Samples meeting minimal quality requirements were then cleaned using either the Qiagen DNeasy PowerClean Pro Cleanup Kit (Qiagen, Hilden, Germany) or Ampure magnetic beads (Agencourt Bioscience Corporation, Beverly, MA). Samples were run on a Qubit prior to normalization. Each sample was run through a test enzyme digestion using mseI restriction enzyme (New England Bio), following the New England Biolabs Optimizing Restriction Endonuclease Reactions protocol, to ensure enzymatic inhibitors were removed during cleaning.

*Sequence processing and bioinformatics*

*Filtering and missing data*

One hundred samples were filtered from the dataset due to a high percentage of missing data. Many of the samples that had high percentages of missing data were older samples (5-10 years old) and were not preserved with liquid nitrogen or cold 95% ethanol, resulting in a smaller quantity of high molecular weight DNA. Multiple filtering approaches were attempted, including removing loci with missing data to attempt to keep more individuals. However, removing loci with high amounts of missing data did not improve the individual sample retention and the approach of filtering out individuals with >90% missing data allowed for the retention of the most samples and loci.

*STRUCTURE*

Within the API toolkit, each population was required to contain a minimum of 25% of its samples and SNPs that were shared across less than 10% of all the samples were excluded. STRUCTURE was run in replicate (n=3, burnin=20,000, numreps = 100,000) using population (*K*) values up to one more than the number of populations (K= 2-10). The results were averaged over the replicates (n=3) for each K value (2-10) and visualized using the package toyplot to assess the likelihood of each K value.  A K=6 was selected as the most likely number of ancestral populations based on the plot below of delatK.


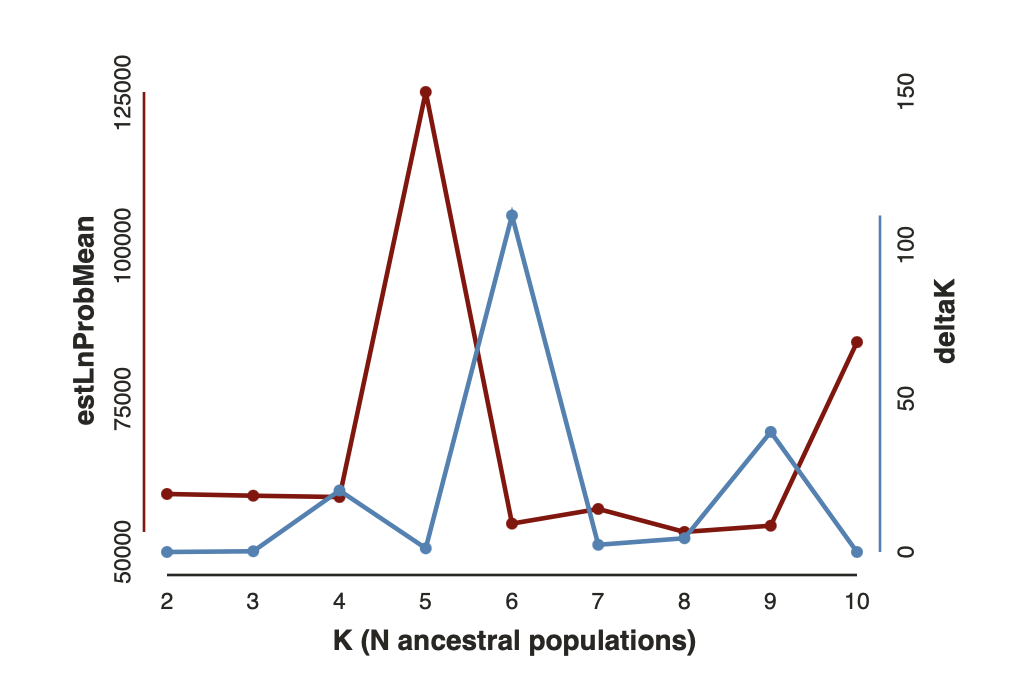

Supplement: Supplementary file 2 — Supplementary Material 2 [file 13104_2024_6977_MOESM2_ESM.docx]
