# Supplementary material for: Population structure of Desmophyllum pertusum found along the United States eastern continental margin
Source: BMC Res Notes. 2024 Oct 29;17:326. doi: 10.1186/s13104-024-06977-4 (PMC11520793; doi:10.1186/s13104-024-06977-4)
Supplement: Supplementary file 3 — Supplementary Material 3 [file 13104_2024_6977_MOESM3_ESM.docx]

Supplementary Figure 1

Title: Population structure of *Desmophyllum pertusum* found along the United States eastern continental margin

Alexis M. Weinnig^1^, Aaron Aunins^1^, Veronica Salamone^1^, Andrea M. Quattrini^2^, Martha S. Nizinski^3,2^, and Cheryl L. Morrison^1^

^1^US Geological Survey, Eastern Ecological Science Center, Leetown Research Laboratory, Kearnesville, WV USA

^2^ Department of Invertebrate Zoology, National Museum of Natural History, Smithsonian Institution, Washington, DC USA

^3^ National Systematics Laboratory, Office of Science and Technology, NOAA Fisheries, Washington, DC USA

**Any use of trade, product, or firm names is for descriptive purposes only and does not imply endorsement by the U.S. Government.**


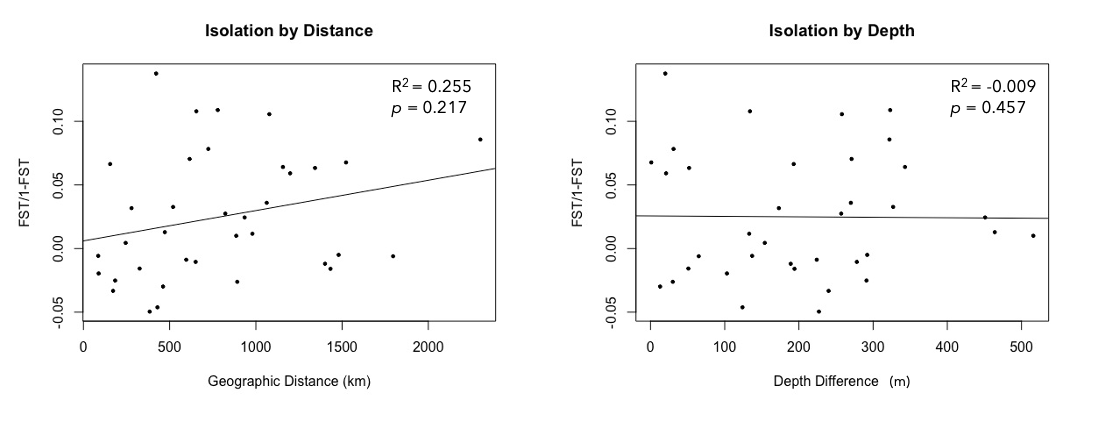


Supplementary Figure 1. Isolation by distance and isolation by depth
