# Supplementary material for: Population structure of Desmophyllum pertusum found along the United States eastern continental margin
Source: BMC Res Notes. 2024 Oct 29;17:326. doi: 10.1186/s13104-024-06977-4 (PMC11520793; doi:10.1186/s13104-024-06977-4)
Supplement: Supplementary file 4 — Supplementary Material 4 [file 13104_2024_6977_MOESM4_ESM.docx]

Supplementary Table 1

Title: Population structure of *Desmophyllum pertusum* found along the United States eastern continental margin

Alexis M. Weinnig^1^, Aaron Aunins^1^, Veronica Salamone^1^, Andrea M. Quattrini^2^, Martha S. Nizinski^3,2^, and Cheryl L. Morrison^1^

^1^US Geological Survey, Eastern Ecological Science Center, Leetown Research Laboratory, Kearnesville, WV USA

^2^ Department of Invertebrate Zoology, National Museum of Natural History, Smithsonian Institution, Washington, DC USA

^3^ National Systematics Laboratory, Office of Science and Technology, NOAA Fisheries, Washington, DC USA

**Any use of trade, product, or firm names is for descriptive purposes only and does not imply endorsement by the U.S. Government.**

**Table 1.** Overall diversity statistics for *Desmophyllum pertusum* samples (n= 57) across 3,191 loci. H_O_ = mean observed heterozygosity, H_E_ = expected heterozygosity or within population gene diversity, H_t_ = overall gene diversity, F_is_ = inbreeding coefficient, and F_ST_ = fixation index

| H_O_ | H_E_ | H_t_ | F_is_ | F_ST_ |
| --- | --- | --- | --- | --- |
| 0.050 | 0.183 | 0.192 | 0.726 | 0.042 |
