# Supplementary material for: Population structure of Desmophyllum pertusum found along the United States eastern continental margin
Source: BMC Res Notes. 2024 Oct 29;17:326. doi: 10.1186/s13104-024-06977-4 (PMC11520793; doi:10.1186/s13104-024-06977-4)
Supplement: Supplementary file 5 — Supplementary Material 5 [file 13104_2024_6977_MOESM5_ESM.docx]

Supplementary Table 2

Title: Population structure of *Desmophyllum pertusum* found along the United States eastern continental margin

Alexis M. Weinnig^1^, Aaron Aunins^1^, Veronica Salamone^1^, Andrea M. Quattrini^2^, Martha S. Nizinski^3,2^, and Cheryl L. Morrison^1^

^1^US Geological Survey, Eastern Ecological Science Center, Leetown Research Laboratory, Kearnesville, WV USA

^2^ Department of Invertebrate Zoology, National Museum of Natural History, Smithsonian Institution, Washington, DC USA

^3^ National Systematics Laboratory, Office of Science and Technology, NOAA Fisheries, Washington, DC USA

**Any use of trade, product, or firm names is for descriptive purposes only and does not imply endorsement by the U.S. Government.**

**Table 2**. Confidence intervals for F-statistics.

|  | H-Total | F-pop/Total | | F-Ind/Total | H-pop | F-Ind/pop | Hobs |
| --- | --- | --- | --- | --- | --- | --- | --- |
| 2.5% | 0.1881 | | 0.0239 | 0.7396 | 0.1773 | 0.7247 | 0.0459 |
| 50% | 0.1942 | | 0.0507 | 0.7527 | 0.1844 | 0.7296 | 0.0480 |
| 97.5% | 0.2003 | | 0.0791 | 0.7644 | 0.1909 | 0.7530 | 0.0504 |
