# Supplementary material for: Population structure of Desmophyllum pertusum found along the United States eastern continental margin
Source: BMC Res Notes. 2024 Oct 29;17:326. doi: 10.1186/s13104-024-06977-4 (PMC11520793; doi:10.1186/s13104-024-06977-4)
Supplement: Supplementary file 6 — Supplementary Material 6 [file 13104_2024_6977_MOESM6_ESM.docx]

Supplementary Table 3

Title: Population structure of *Desmophyllum pertusum* found along the United States eastern continental margin

Alexis M. Weinnig^1^, Aaron Aunins^1^, Veronica Salamone^1^, Andrea M. Quattrini^2^, Martha S. Nizinski^3,2^, and Cheryl L. Morrison^1^

^1^US Geological Survey, Eastern Ecological Science Center, Leetown Research Laboratory, Kearnesville, WV USA

^2^ Department of Invertebrate Zoology, National Museum of Natural History, Smithsonian Institution, Washington, DC USA

^3^ National Systematics Laboratory, Office of Science and Technology, NOAA Fisheries, Washington, DC USA

**Any use of trade, product, or firm names is for descriptive purposes only and does not imply endorsement by the U.S. Government.**

**Table 3**. Population level statistics for nine *Desmophyllum pertusum* sample sites. N = number of individuals, Ho = observed heterozygosity, He = expected heterozygosity or within population gene diversity, and Fis = inbreeding coefficient

| Sample Site | N | Mean H_O_ | Mean H_E_ | Mean F_is_ |
| --- | --- | --- | --- | --- |
| New England Intercanyons | 7 | 0.04 | 0.16 | 0.596 |
| Richardson Reef | 22 | 0.05 | 0.18 | 0.567 |
| Cape Fear | 4 | 0.05 | 0.14 | 0.456 |
| Savanah Banks | 3 | 0.07 | 0.15 | 0.413 |
| Stetson Bank | 2 | 0.03 | 0.11 | 0.589 |
| Canaveral | 4 | 0.06 | 0.15 | 0.440 |
| Pea Island | 1 | 0.04 | Na | Na |
| Norfolk Canyon | 5 | 0.06 | 0.12 | 0.383 |
| Gulf of Mexico | 9 | 0.04 | 0.14 | 0.596 |
